# The impact of physical exercise on convergent and divergent thinking

**DOI:** 10.3389/fnhum.2013.00824

**Published:** 2013-12-02

**Authors:** Lorenza S. Colzato, Ayca Szapora, Justine N. Pannekoek, Bernhard Hommel

**Affiliations:** ^1^Cognitive Psychology Unit, Institute for Psychological Research and Leiden Institute for Brain and Cognition, Leiden UniversityLeiden, Netherlands; ^2^Department of Psychiatry and Mental Health, University of Cape TownCape Town, South Africa; ^3^Leiden University Medical Centre and Leiden Institute for Brain and Cognition, Leiden UniversityLeiden, Netherlands

**Keywords:** physical exercise, creativity, convergent thinking, divergent thinking, fitness

## Abstract

Anecdotal literature suggests that creative people sometimes use bodily movement to help overcome mental blocks and lack of inspiration. Several studies have shown that physical exercise may sometimes enhance creative thinking, but the evidence is still inconclusive. In this study we investigated whether creativity in convergent- and divergent-thinking tasks is affected by acute moderate and intense physical exercise in athletes (*n* = 48) and non-athletes (*n* = 48). Exercise interfered with divergent thinking in both groups. The impact on convergent thinking, the task that presumably required more cognitive control, depended on the training level: while in non-athletes performance was significantly impaired by exercise, athletes showed a benefit that approached significance. The findings suggest that acute exercise may affect both, divergent and convergent thinking. In particular, it seems to affect control-hungry tasks through exercise-induced “ego-depletion,” which however is less pronounced in individuals with higher levels of physical fitness, presumably because of the automatization of movement control, fitness-related neuroenergetic benefits, or both.

## INTRODUCTION

Anecdotal literature suggests that creative people sometimes use bodily movement to help overcome mental blocks and to get deeper into a problem. Indeed, the philosopher Henry David Thoreau stated: “the moment my legs begin to move my thoughts begin to flow – as if I had given vent to the stream at the lower end and consequently new fountains flowed into it at the upper” (Thoreau, 1851). Several studies have indeed shown that physical exercise in healthy adults may sometimes enhance creative thinking – even though the size of this effect can vary substantially (Gondola and Tuckman, 1985; Gondola, 1986, 1987; Steinberg et al., 1997; Blanchette et al., 2005). Gondola and Tuckman (1985) investigated the effects of long-term physical exercise on creativity performance, showing small but significant improvements in Alternate Uses (spontaneous flexibility) and Remote Consequences (originality) tasks, but not for an Obvious Consequences (different ideas) task. Gondola (1986) used the same creativity tasks to compare the effect of long-term and acute physical exercise and found improvements for both conditions and all three creativity measures. Gondola (1987) tested another form of acute aerobic activity (dance) and reported comparable enhancing effects. Steinberg et al. (1997) found only small improvements in a group of fit participants, and only in one of the three measures of the Torrance test of creative thinking. Blanchette et al. (2005) used the same test and found enhancing effects of exercise over a 2 h period. It is possible that in some or all of these previous studies physical exercise provided the opportunity for mind-wandering or incubation in trained (and, thus, less challenged) people. Indeed, Baird et al. (2012) have reported that engaging in simple external tasks that allow the mind to wander may facilitate creative problem solving.

The methodological diversity across the available studies with regard to sample characteristics and creativity assessment (mainly targeting aspects of divergent thinking) is considerable, which renders it questionable whether they were actually assessing the same constructs and processes. Moreover, there is still no mechanistic model explaining how creative processes operate and how physical exercise might affect these operations. To address this issue, we tried to avoid addressing creativity as a whole but focused on particular components of creative performance – components that are more transparent at the process level and thus easier to investigate. More concretely, we investigated the impact (during and after) of acute moderate and intense physical exercise on creativity tasks tapping into convergent and divergent thinking. Guilford (1950, 1967) has considered these two as the main ingredients of most creative activities, even though other processes are also likely to contribute (Wallas, 1926).

Divergent thinking is taken to represent a style of thinking that allows many new ideas being generated, in a context where more than one solution is correct. The probably best example is a brainstorming session, which has the aim of generating as many ideas on a particular issue as possible. Guilford’s (1967) alternate uses task (AUT) to assess the productivity of divergent thinking follows the same scenario: participants are presented with a particular object, such as a pen, and they are to generate as many possible uses of this object as possible. Convergent thinking, in turn, is considered a process of generating one possible solution to a particular problem. It emphasizes speed and relies on high accuracy and logic. Mednick’s (1962) remote associates task (RAT) that aims to assess convergent thinking fits with this profile: participants are presented with three unrelated words, such as “time,” “hair,” and “stretch,” and are to identify the common associate (“long”). Interestingly for our purposes, performance on the AUT and the RAT were found to be uncorrelated (Akbari Chermahini and Hommel, 2010) and differently affected by the same experimental manipulations (Hommel et al., submitted), which supports Guilford’s (1967) suggestion that convergent and divergent thinking represent different, separable components of human creativity. Such a scenario would fit with considerations of De Dreu et al. (2008), who proposed the Dual Pathway to Creativity model suggesting that creative performance arises from the interaction between cognitive flexibility and cognitive persistence – two dissociable cognitive control functions (Goschke, 2000; De Dreu et al., 2012). Consistent with this, divergent thinking was less pronounced in avoidance-motivated than in approach-motivated individuals, suggesting that the former need to compensate for their inflexible processing style by effortful and controlled processing (Roskes et al., 2012).

Along the same lines, Colzato et al. (2012) have argued that convergent thinking requires strong top-down control because it represents the tightly constrained search of very few or just one item. In contrast, divergent thinking should rely on weak top-down control, given that it implies a broad, loosely defined search space so to activate many items that satisfy the often relatively soft criteria (Hommel, 2012). Hence, convergent and divergent thinking are likely to differ in their reliance on executive control for the processing of information. If so, acute exercise should affect these two processes differently. According to the ego-depletion hypothesis (Baumeister et al., 1998), the cognitive resources required for cognitive-control operations are tightly limited and thus deplete quickly during and after control-demanding tasks. Following a similar, though more motivational rationale, Inzlicht and Schmeichel (2012) have developed a process model to explain self-control failure. According to that model, “exerting self-control at Time 1 reduces success at self-control at Time 2 by initiating shifts in motivation and attention that conspire to reduce self-control and increase immediate gratification” (p. 460). According to this reasoning, poorer self-control at Time 2 is attributed to reduced motivation to exert control and to reduced attention to cues that signal a need for control, as well as more impulsive behavior and more attention to reward cues. Given that exercising must use up some amount of control resources, more control-demanding tasks (like convergent thinking) should suffer more from exercise than less control-demanding tasks (like divergent thinking).

However, how resource-hungry exercise should not only depend on the kind of exercise (e.g., the complexity of the coordination required) but also on the skill level of the exercising individual. The same exercise that exhausts the resources of the less sportive student may have little impact on the highly practiced athlete. In athletes, many movement routines are overlearned and automatized, which can lead to dramatic reductions of conscious monitoring and control demands (Beilock and Carr, 2001; Schneider and Chein, 2003). Moreover, long-term fitness training leads to an increase of oxygenation and glucose in the frontal brain regions, which has been found to produce rather selective benefits for executive-control processes (Colcombe and Kramer, 2003). This means that athletes may not exhibit the same effects as non-athletes. While the latter should show exercise-induced costs in more control-demanding tasks (like convergent thinking), the former might either not show such costs or perhaps even show exercise-induced benefits.

To investigate these possibilities, we tested the impact of acute physical exercise on convergent and divergent thinking in athletes and non-athletes. We also took into account possible moderating factors, such as the intensity of the exercise (which was moderate or high, in different sessions) and the temporal overlap between exercise and creativity task (with the latter being performed during or after the exercise).

## METHODS

### PARTICIPANTS

Ninety-six healthy, native Dutch speakers (48 females and 48 males), of which 48 were athletes (mean age = 20.6 years; mean body mass index, BMI = 22.3) and 48 non-athletes (mean age = 20.7 years; mean BMI = 22.2), participated for an energy bar and a sports drink or one study credit. Participants were considered athletes if they exercised at least three times a week during the recent 2 years and non-athletes if they did not exercise on a regular basis (less than 1 time per week). All participants had normal systolic and diastolic blood pressure at rest (mean systolic blood pressure, SBP = 122 and diastolic blood pressure, DPB = 74), and reported no current or history of medication or drug use. Informed consent was obtained from all participants after the nature of the study was explained to them. The protocol was approved by the local ethical committee (Leiden University, Institute for Psychological Research).

### REMOTE ASSOCIATION TASK (CONVERGENT THINKING)

In this task, participants are presented with three unrelated words (such as “time,” “hair,” and “stretch”) and asked to find a common associate (“long”). Our Dutch version comprised of 30 previously validated items (Akbari Chermahini et al., 2012). In each of the three sessions, participants completed 10 different items.

### ALTERNATE USES TASK (DIVERGENT THINKING)

In this task, participants were asked to list as many possible uses for six common household items (“pen,” “towel,” “bottle”). In the three sessions, participants completed 1 of these items. The results can be scored in several ways with *flexibility*, the number of different categories used, being the theoretically most transparent and the empirically most consistent and reliable score (Akbari Chermahini and Hommel, 2010). In the case of the item “pen,” “writing an essay,” and “writing a letter” would fall into the same category, but “drumming on the table” would fall into a different category.

In this study we considered four scores:

*Flexibility:* The number of different categories used.

*Originality:* Each response is compared to the total amount of responses from all of the subjects. Responses that were given by only 5% of the group count as unusual (1 point) and responses given by only 1% of them count as unique (2 points).

*Fluency:* The total of all responses.

*Elaboration:* The amount of detail (e.g., “a door stop” counts 0, whereas “a door stop to prevent a door slamming shut in a strong wind” counts 2 (1 point for explanation of door slamming and another for further detail about the wind).

### EXERCISE CONDITIONS

During the rest condition, participants sat on a cycle ergometer (Kettler Cycle) without cycling. During the moderate cycling condition, participants cycled at a normal pace (level 8) without exhausting themselves. During the intense cycling condition, the resistance level on the bicycle was adjusted to high (level 16), and the participants cycled at a maximum level of effort.

### PHYSIOLOGICAL AND MOOD MEASUREMENTS

Heart rate (HR) and systolic and diastolic blood pressure (SBP and DPB) were measured from the non-dominant arm with an OSZ 3 Automatic Digital Electronic Wrist Blood Pressure Monitor (Speidel and Keller). BMI was measured by Omron BF511 medical device. Mood was rated on a 9 × 9 Pleasure × Arousal grid (Russell et al., 1989) with values ranging from –4 to 4.

### PROCEDURE AND DESIGN

A between-group (athletes vs. non-athletes) randomized cross-over design with counterbalancing of the order of the exercise conditions (rest vs. moderate vs. intense) was used (Latin-square design). All participants were tested individually. Half of the participants in each group (*n* = 24) executed the creativity tasks during cycling, the other half (*n* = 24) thereafter. Upon arrival, participants were asked to rate their mood and HR, SBP, DPB, and BMI were collected (baseline measurement). Next, the participant was introduced to the assigned exercise condition. When the rest condition was preceded by the moderate or intense exercise condition, the participant started the next exercise condition only after a couple of minutes (never more than 5) when HR returned to the baseline measurement level.

**FIGURE 1:**
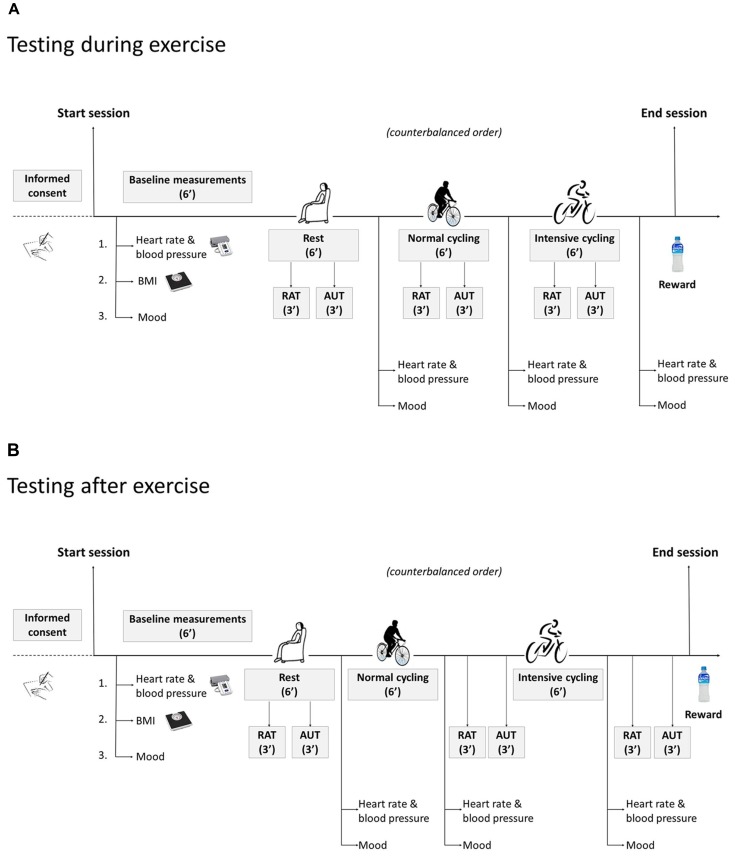
**Sequence of events for the participants who performed the creativity tasks during exercise (A)** or after exercise **(B).**

After each condition, HR, SBP, DPB, and mood were measured again. The creativity tasks (AUT and RAT) were performed either during or after the physical exercise, depending on the condition subjects had been randomly assigned to, see **Figure 1**. Participants had 3 min to execute the RAT (10 items per test condition) and 3 min for the AUT (1 item per test condition). Participants were confronted with a printed version of the creativity tasks on a clipboard positioned on the cycle ergometer in front of them so that they could fill in their responses comfortably while cycling. After the experimental session was ended, participants were rewarded for their participation in the study.

### STATISTICAL ANALYSIS

Independent *t*-tests were performed to test differences between the two groups. Mood, HR, BPS, and BPD, and five creativity measures (from the two tasks) were extracted for each participant: flexibility, originality, fluency, and elaboration scores from the AUT, the number of correct items from the RAT. All four AUT measures were scored by two independent raters [Cronbach’s alpha = 1.00 (fluency); 0.85 (flexibility); 0.71 (originality); 0.74 (elaboration)]. All measures were analyzed separately by means of repeated-measures ANOVAs with Session (rest vs. normal vs. intense) as within-subjects factor and group (athletes vs. non-athletes) and moment in which participants carried out the creativity tasks (during vs. after exercise) as between-group factor. A significance level of *p* < 0.05 was adopted for all tests.

## RESULTS

### PARTICIPANTS

No significant group differences were obtained for age, *t*(94) = 0.05, *p* = 0.95, and BMI, *t*(94) = 0.34, *p* = 0.73, but there was a significant difference for sport units per week, *t*(94) = 21.68, *p* = 0.00001: athletes exercised more often per week (3.4) than non-athletes did (0.5).

### PHYSIOLOGICAL AND MOOD MEASUREMENTS

We found a main effect of session on HR, *F*(2,184) = 768.01, *p* < 0.00001, MSE = 109.063, *η2p* = 0.89, SBP, *F*(2,184) = 165.76, *p* < 0.00001, MSE = 163.793, *η2p* = 0.64, and DBP, *F*(2,184) = 29.18, *p* < 0.001, MSE = 104.509, *η2p* = 0.24. Participants showed increased HR, SBP, and DBP in the moderate (95, 130, 76) and intense (133, 150, 85) exercise condition as compared to the rest condition (75, 116, 74). No other significant interaction involving group was found, *p* > 0.14.

Replicating earlier findings (Steptoe and Bolton, 1988), arousal, *F*(2,184) = 768.01, *p* < 0.00001, MSE = 109.063, *η2p* = 0.89, but not mood, *F*(2,184) = 43.71, *p* < 0.0001, MSE = 1.077, *η2p* = 0.32, was elevated after intense exercise (1.9, 1.1) as compared to normal exercise (1.1, 1.3) and rest (0.6, 1.2), respectively. As in the case of physiological measurements, no other significant interaction involving group was found, *F* < 1.

### CREATIVITY TASKS

In general, performance in the AUT and RAT was good and comparable to performance in other studies without exercise manipulations (e.g., Akbari Chermahini and Hommel, 2010); see **Table 1**.

**Table 1:** Means for the number of correct items from the remote associates task (RAT), for flexibility, originality, fluency, and elaboration scores from the alternate uses task (AUT), and perceived mood ratings as a function of group (athletes vs. non-athletes), session (rest vs. normal vs. intense) and moment in which participants carried out the creativity tasks (during vs. after exercise).

Group	Moment	Session	RAT	AUT-flexibility	AUT-originality	AUT-fluency	AUT-elaboration	HR	BPS	BPD	Mood	Arousal
Athletes	During	Rest	3.6	7.3	0.50	11.0	0.83	77.0	113.5	74.4	1.5	0.7
		Normal	3.9	6.7	0.79	11.0	0.67	94.4	127.9	74.8	1.9	1.3
		Intense	4.0	6.2	0.75	10.5	0.62	126.1	148.6	83.1	1.8	2.0
	After	Rest	3.5	6.9	0.83	11.1	0.96	71.9	116.9	71.5	1.2	0.2
		Normal	4.3	6.7	0.79	10.8	0.87	91.0	134.8	74.6	1.1	1.1
		Intense	4.3	6.8	0.70	10.8	0.96	134.8	151.5	83.1	0.8	1.7
Non-athletes	During	Rest	4.7	7.2	0.46	10.4	0.92	75.5	117.5	77.2	1.2	0.6
		Normal	4.8	6.4	0.50	9.2	0.79	93.2	130.6	76.4	0.9	1.1
		Intense	3.4	6.7	0.37	8.6	0.62	131.6	150.8	88.1	0.9	2.0
	After	Rest	4.5	7.9	0.54	10.6	1.04	76.0	117.2	74.3	0.9	0.8
		Normal	4.0	7.9	0.46	11.0	1.00	102.8	127.3	79.2	1.5	0.8
		Intense	3.9	7.1	0.42	10.2	0.96	140.7	148.0	85.5	0.9	1.8

Convergent thinking: As expected, we found a significant interaction between group and session on RAT scores, *F*(2,184) = 5.16, *p* < 0.01, MSE = 2.838, *η2p* = 0.05. *Post-hoc* multiple comparisons tests revealed that, even if not quite significant, athletes tended to perform better in convergent thinking in the moderate (4.1) and intense (4.2) exercise conditions than in the rest condition (3.5), *p* = 0.072, 0.095. This effect was reversed in non-athletes, where intense exercise (3.6) impaired convergent thinking compared to moderate exercise (4.4), *p* = 0.002 and rest (4.6), *p* = 0.029. The interaction was not modified by testing moment, as the insignificant three-way interaction indicated, *F*(2,184) = 1.01, *p* = 0.364, MSE = 2.838, *η2p* = 0.01.

Divergent thinking: From the four scores of the AUT, only flexibility yielded a significant main effect of session, *F*(2,184) = 3.69, *p* < 0.05, MSE = 3.169, *η2p* = 0.03; *post-hoc* tests revealed that participants showed greater flexibility in the rest condition (7.4) than with intense (6.7) exercise, *p* = 0.011, while the difference between rest and moderate exercise (7.0) only approached significance, *p* = 0.150. Numerically similar, but statistically insignificant trends were obtained for originality, *F*(2,184) = 0.42, *p* = 0.66, MSE = 0.320, *η2p* = 0.05, fluency, *F*(2,184) = 2.47, *p* = 0.09, MSE = 5.420, *η2p* = 0.03, and elaboration, *F*(2,184) = 2.19, *p* = 0.11, MSE = 0.247, *η2p* = 0.02. In contrast to the RAT findings, the flexibility effect was not modulated by group, *F* < 1, and the same was true for originality, *F*(2,184) = 1.20, *p* = 0.302, MSE = 0.320, *η2p* = 0.01, fluency, *F* < 1, and elaboration, *F*(2,184) = 1.07, *p* = 0.346, MSE = 2.838, *η2p* = 0.01. There was also no indication of any three-way interaction, *p*’s **> 0.21.

## DISCUSSION

In this study we investigated whether creativity in convergent- and divergent-thinking tasks is affected by acute physical exercise. The results provide some preliminary evidence for a link between exercise and creativity, but they also suggest that the nature and the consequences of this link depend on the particular task and the fitness of the individual.

First, non-athletes did not benefit from acute exercise; in fact, exercise caused their performance to drop in both creativity tasks. The fact that this drop was not modified by the moment of testing suggests that it was not due to dual-tasking or related online demands. Rather, in this group acute exercise seems to lead to ego-depletion, hence, exhaust limited cognitive-control resources (Baumeister et al., 1998) that are then no longer available for the control of processes involved in convergent and divergent thinking. Future research needs to clarify whether there is something specific about physical exercise that depletes cognitive resources over and above the complexity of the exercise. In particular, it would be important to determine whether depletion reflects the physical aspect of exercise or the cognitive demand.

Second, athletes tended to benefit from acute exercise in the convergent-thinking task. While this benefit was not quite reliable, we may speculate that athletes are shielded from the exercise-induced cognitive costs that non-athletes exhibited. This shielding effect is likely to reflect one or both of two possibilities. For one, athletes may have developed more automatic action-control routines, which frees capacity-limited processes from engaging and action monitoring and control (Beilock and Carr, 2001; Schneider and Chein, 2003). If so, the exercise might have been less control-hungry and capacity demanding in our athletes than in the non-athletes, so that more control capacity was left for the convergent-thinking task. Further testing this possibility would require a conceptual framework that allows determining the resource overlap between exercise and cognitive task pre-experimentally, and which allows predictions regarding the kind of resource that can be saved through automatization. For another, the shielding effect seems to fit with the idea that physical exercise, and the resulting increase of oxygenation and glucose in frontal brain regions, prevents or at least works against exercise-related ego-depletion. It is also partially in line with Colcombe and Kramer’s (2003) consideration that aerobic fitness training might lead to the enhancement of cognitive-control processes and tasks relying on them. Even though our data do not show reliable enhancement, it is true that our criterion for categorizing participants as athletes was relatively modest. Hence, it is not unreasonable to suspect that even more active individuals do show reliable benefits in tasks relying on convergent thinking. However, athletes performed worse on the RAT than non-athletes in the rest condition. It is not to exclude that the enhancement of cognitive-control processes by aerobic fitness is so short-lived that positive effects are restricted to performance during or directly after exercising. From the current results, one may even speculate that for people who are used to exercise, the absence of exercise (rest) impairs (creative) performance more than its presence improves it. More generally, performance may be best whenever one carries out one’s preferred (non-)activity.

Third, we sought to characterize the relationship between creativity and physical exercise by investigating the impact of two potential moderators: the intensity of the exercise and the temporal overlap between exercise and cognitive task. Whereas the latter factor did not seem to have any measurable impact, which rules out an account of our findings in terms of technical or motor problems (e.g., motor interference when responding to the items), the former does: intense exercise seems to enhance performance in athletes (at least numerically) and to impair performance in non-athletes the most. This opens the possibility to use more parametric manipulations of exercise and the possibly resulting ego-depletion to investigate both positive and negative exercise effects. In any case, future research needs to replicate and extend the present observations.

Even though we found more evidence for negative than for positive effects, our observations suggest that more exercise may enhance convergent thinking, at least in individuals with a higher degree of physical fitness. We should point out that there was no main effect of fitness, in the sense that athletes outperformed non-athletes in convergent thinking as such. However, given that we did not manipulate fitness experimentally, this may very well be an artifact of self-selection. Testing this possibility would require more extended studies in which physical fitness is under direct experimental control. It would also require more consideration of the role of individual differences, especially with respect to preexisting neuro-developmental factors. Such differences may affect the degree to which individuals can benefit from fitness training: individuals with a certain genetic predispositions may take advantage from a given type of training whereas individuals with another predisposition may not. It would also be important to include other physiological measures such as volumes of oxygen to further investigate the neural mechanism by which exercise may affect creativity.

## Conflict of Interest Statement

The authors declare that the research was conducted in the absence of any commercial or financial relationships that could be construed as a potential conflict of interest.
